# Lack of association between gene polymorphisms of *Angiotensin converting enzyme*, *Nod-like receptor 1*, *Toll-like receptor 4, FAS/FASL *and the presence of *Helicobacter pylori*-induced premalignant gastric lesions and gastric cancer in Caucasians

**DOI:** 10.1186/1471-2350-12-112

**Published:** 2011-08-24

**Authors:** Juozas Kupcinskas, Thomas Wex, Jan Bornschein, Michael Selgrad, Marcis Leja, Elona Juozaityte, Gediminas Kiudelis, Laimas Jonaitis, Peter Malfertheiner

**Affiliations:** 1 Department of Gastroenterology, Lithuanian University of Health Sciences, Eiveniu 2, 50009 Kaunas, Lithuania; 2 Clinic of Gastroenterology, Hepatology and Infectious Diseases, Otto von Guericke University, Leipziger Str. 44, 39120 Magdeburg, Germany; 3 Faculty of Medicine, University of Latvia, Digestive Diseases Center, Hospital Lizeners, 6 Linezera iela, LV1006 Riga, Latvia; 4 Department of Oncology, Lithuanian University of Health Sciences, Eiveniu 2, 50009 Kaunas, Lithuania

## Abstract

**Background:**

Several polymorphisms of genes involved in the immunological recognition of *Helicobacter pylori *and regulating apoptosis and proliferation have been linked to gastric carcinogenesis, however reported data are partially conflicting. The aim of our study was to evaluate potential associations between the presence of gastric cancer (GC) and high risk atrophic gastritis (HRAG) and polymorphisms of genes encoding *Angiotensin converting enzyme *(*ACE*), *Nod-like receptor 1 *(*NOD1*), *Toll-like receptor 4 *(*TLR4*) and *FAS/FASL*.

**Methods:**

Gene polymorphisms were analyzed in 574 subjects (GC: n = 114; HRAG: n = 222, controls: n = 238) of Caucasian origin. *ACE I/D *(rs4646994), *NOD1 796G>A *(rs5743336), *TLR4 3725G>C *(rs11536889), *FAS 1377G>A *(rs2234767), *FAS 670A>G *(rs1800682) and *FASL 844T>C *(rs763110) were genotyped by different PCR approaches and restriction fragment length polymorphism analysis.

**Results:**

Frequencies of genotypes in our study are similar to the data reported on subjects of Caucasian ethnicity. There was a tendency for *NOD1 796G/G *genotype to be associated with increased risk of HRAG (62.4% *vs*. 54.5% in controls, *p *= 0.082). *FAS 670G/G *genotype was more frequent in HRAG when compared to controls, 23.9% and 17.2% respectively, however it failed to reach significance level (*p *= 0.077). We did not find any significant associations for all polymorphisms in relation to GC or HRAG. *NOD1 796G>A *and *TLR4 3725G>C *gene polymorphisms were also not associated with *Helicobacter pylori *infection.

**Conclusions:**

*ACE, NOD1, TRL4 *and *FAS/FASL *gene polymorphisms are not linked with gastric carcinogenesis in Caucasians, and therefore they should not be considered as potential biomarkers for identifying individuals with higher risk for GC.

## Background

Based on the current paradigm, gastric carcinogenesis is considered as multistep process involving complex interplay between *Helicobacter pylori *(*H. pylori*) infection, environmental and host genetic factors [1]. In gastric carcinogenesis two distinct pathways have been suggested for intestinal and diffuse type of gastric cancer (GC) (1). Correa *et al*. [2] demonstrated that *H. pylori *infection induces chronic inflammation of the gastric mucosa, leading to atrophic gastritis (AG) and intestinal metaplasia (IM), which are regarded as essential predisposing factors in intestinal-type GC development. Many researchers have shown a close relationship between *H. pylori *not only for intestinal-type GC, but also for diffuse-type GC [3,4]. Furthermore, although AG and IM are considered as obligatory predisposing factors in intestinal-type GC, the accumulating evidence suggests that AG and IM are at least partially associated with GC of the diffuse-type as well [3]. Up to date, no molecular screening methods are available for sporadic GC, therefore pathogenetic mechanisms involved in different stages of gastric carcinogenesis need to be further elucidated to allow identification of individuals with increased risk for GC. Recent studies have revealed several genetic polymorphisms related to immune-recognition of *H. pylori*, cell proliferation, and apoptosis pathways that were linked with premalignant gastric conditions and GC, but further research is needed to establish their role in gastric carcinogenesis [5].

*Angiotensin I-converting enzyme *(*ACE*) is expressed by different cell types [6] and generates *Angiotensin II*, which is a key effector in renin-angiotensin system. Recent reports provided evidence that *Angiotensin II *is involved in the regulation of cell proliferation, angiogenesis and inflammation through the *Angiotensin II type 1 receptors*, which are expressed on tumor and endothelial cells [7-9]. Insertion (*I*) or deletion (*D*) polymorphism of *ACE *gene was shown to have functional relevance, since the carriers of *D *allele have higher *ACE *activity [10]. *ACE I/D *polymorphism was linked with early development and spread of GC [11], but the studies report null association with the overall risk for GC [12,13]. *ACE I/D *polymorphism was not linked with gastric atrophy in Asian subjects [14]. There are however no reports that evaluate the role *ACE I/D *polymorphism in premalignant gastric conditions in Caucasians and studies addressing the role of ACE I/D polymorphisms in gastric carcinogenesis are limited.

*Nucleotide-binding oligomerisation domain 1 *(*NOD1*) is a member of the Nod-like receptors, which is expressed in the cytoplasm of antigen presenting cells and gastric epithelial cells and is involved in recognition of gram-negative bacteria [15]. It is known that stimulation of gastric epithelial cells with *NOD1 *ligands leads to production of proinflammatory cytokines [16,17] and *NOD1 *participates in host defense against mucosal infection with *H. pylori *infection [17,18]. Recently *NOD1 *was found to respond to peptidoglycan delivered by *H. pylori cag*PAI [19]. Given the significant role of *H. pylori *in gastric carcinogenesis, it is hypothesized that genetic variations in gene encoding *NOD1 *receptor could be related to different outcomes. Gene polymorphism *796G>A *of *NOD1 *has been linked with peptic ulcer disease in *H. pylori*-positive patients [20] and a significant association with very high odds ratios has been recently reported for the risk of premalignant lesions in the antrum of the stomach [21]. Up to date, the data on *NOD1 796G>A *gene polymorphism remain very scarce and there are no reports concerning the role of this polymorphism in patients with GC.

Another important receptor for the recognition of *H. pylori *is *Toll-like receptor 4 *(*TLR4*), a member of Toll-like receptors family. *TLR4 *recognizes lipopolysaccharide of gram-negative bacteria, and it is thought to interact with macrophage/monocyte in response to *H. pylori *infection [22]. Expression of *TLR4 *by gastric epithelium is upregulated in *H. pylori-*induced gastritis compared to non-inflamed gastric mucosa [23]. Single nucleotide polymorphisms (SNPs) of *TLR4 *gene are thought to disrupt the normal structure of the extracellular region of the *TLR4 *and are therefore hypothesized to decrease responsiveness to lipopolysaccharide through alterations in binding. *TLR4 896A>G *polymorphism was found to be associated with higher risk of *H. pylori*-induced GC and its precursors [24]. Recently, a novel polymorphism in *TLR4 *gene *3725G>C *(rs11536889) with functional relevance was identified [25] and linked with severe gastric atrophy in Asian population [26]. This is however the only report on *TLR4 3725G>C *polymorphism and the potential link with premalignant gastric lesions or GC has not been evaluated yet.

The role of apoptosis in tumor genesis has been well established [27]. The *FAS *and *FASL *system plays an important role in regulating apoptotic cell death, initiating the extrinsic pathway of apoptosis [28]. Decreased expression levels of *FAS *and *FASL *are associated with different malignancies as well as the progression of gastric carcinoma [29]. Several single nucleotide polymorphisms (SNPs) *FAS 1377G>A *(rs2234767), *FAS 670A>G *(rs1800682) and *FASL 844T>C *(rs763110) were shown to affect gene expression [30,31]. Liu *et al*. showed an association between these *FAS/FASL *gene polymorphisms and higher risk of GC [32], while other studies reported null associations [33,34]. These polymorphisms were also associated with the risk of atrophic gastritis [35], however the data published on the association with risk for GC are still scarce and there are no reports on the role FAS/FASL polymorphisms with respect to GC in Caucasians.

Given the pathophysiological significance of *ACE*, *NOD1*, *TLR4*, *FAS *and *FASL *genes in gastric carcinogenesis, it is intriguing to assess the role of these polymorphisms for the development of GC and *H. pylori*-associated premalignant gastric lesions. Therefore, the aim of our study was to evaluate potential links between the presence of GC or HRAG and *ACE I/D, NOD1 796G>A, TLR4 3725G>C, FAS 1377G>A, FAS 670A>G *and *FASL 844T>C *gene polymorphisms in 114 patients with GC, 222 patients with HRAG and 238 controls of Caucasian origin. This study provides further insights in the puzzle of genetic susceptibility for GC and its precursors.

## Methods

### Study population

Subjects included in the study came from our previous research groups on *IL-1B*, *IL-1RN *and *NOD2 *gene polymorphisms [36-38]. Patients were recruited at three gastroenterological centers in Germany, Lithuania and Latvia. In Germany, patients with GC, HRAG and healthy controls were recruited from the Department of Gastroenterology of the Otto-von-Guericke University Magdeburg between 1998 and 2008. Patients with HRAG were included from the Out-patient Department, and from a clinical study aimed at the long-term follow up of *H. pylori *infection. Controls were recruited from clinical studies with healthy volunteers and subjects from the Out-patient Department with dyspeptic symptoms.

In Lithuania and Latvia, patients with HRAG and controls were included from the Out-patient Departments of University Hospitals in Kaunas and Riga. All the individuals were referred for upper endoscopy because of dyspeptic symptoms during the period of 2005-2006.

The inclusion criteria of HRAG and controls were no history of malignancy, gastrointestinal disease or surgery. GC was determined by histology. From all participants, DNA for genotyping was available. In total, 346 individuals (114 GC, 140 HRAG, 92 controls) from Germany and 228 individuals from Lithuania and Latvia (82 HRAG, 146 controls) were included. All patients were of Caucasian ethnicity. The study was approved by the Ethics Committees of the OvG University Magdeburg, Lithuanian University of Health Sciences and University of Latvia, and informed consent to participate in the study was obtained from all subjects included.

### Histological analysis and *H. pylori *status

Scoring of atrophic gastritis and intestinal metaplasia was done according to the modified Sydney classification [39]. Histological evaluation for GC type was carried out according to the Laurén classification [40]. HRAG was defined as pan-gastritis (similar inflammatory scores in antrum and corpus), corpus-predominant gastritis with or without the presence of gastric atrophy, and intestinal metaplasia either in antrum or corpus as described by Uemura *et al*. and Meining *et al*. [41,42]. *H. pylori *status was determined by testing for anti-*H. pylori IgG *antibodies in sera.

### Genotyping

Genomic DNA was extracted from peripheral blood mononuclear cells using the QIAamp DNA blood kit (Qiagen, Hilden, Germany) according to the manufacturer's instructions.

#### ACE I/D

**Figure 1:**
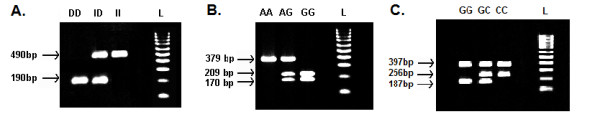
**Gene polymorphisms were determined by DNA fragment migration profile on agarose gel and visualized by ethidium bromide staining: **A**. *ACE I/D *genotypes: *D *or *I *alleles were identified by the presence of 190 or 490 bp fragments **B**. *NOD1 796G>A *genotypes: wild-type DNA is visible as a double-band 209 bp and 170 bp (*GG*); the mutated DNA is visible as a single 379 bp band (*AA*); heterozygotes give three bands (*GA*); **C**. *TLR4 3725G>C *genotypes: wild-type is visible as a double-band 184 and 397 bp (*GG*), mutated DNA is visible as 256 and 397 bp bands (*CC*); heterozygotes give three bands 184, 256, and 397 bp bands (*GC*)**. Lane L is a 100 bp molecular weight marker (HyperLadder IV, Bioline GmbH, Berlin, Germany).

The *ACE I/D *genotype **(**rs4646994) was determined by PCR using primers: forward 5'-CTGGAGACCACTCCCATCCTTTCT-3' and reverse, 5'-GATGTGGCCATCACATTCGTCAGAT-3'. An initial 15 minute denaturation at 95° was followed by 40 cycles of 1 min at 64°C, 1 min at 72°C, and 35 s at 95.5°C. Amplified *ACE *gene polymorphisms were separated on 1.0% agarose gels, and visualized by ethidium bromide staining. *D *and *I *alleles were determined by the presence of 190 or 490 bp fragments (Figure 1).

#### NOD1 796G>A

The *NOD1 796G>A *SNP (rs5743336) was analyzed by PCR restriction fragment length polymorphism analysis (PCR-RFLP) with the following primers: forward 5'-TGA GAC CAT CTT CAT CCT GG-3'; reverse 5'-CTT CCC ACT GAG CAG GTT G-3'. An initial 15 minute denaturation at 95° was followed by 40 cycles of 1 min at 64°C, 1 min at 72°C, and 35 s at 95.5°C. For RFLP analysis, the PCR products were digested with *Ava*I restriction enzyme (Fermentas, Vilnius, Lithuania) at 37°C overnight, studied by gel electrophoresis on a 2% agarose gel and visualized with ethidium bromide staining. The presence of *G *allele was indicated by cleavage of the 379 bp amplified PCR product to yield fragments of 209 bp and 170 bp (Figure 1).

#### TLR4 3725G>C

The *TLR4 3725G>C *SNP (rs11536889) was genotyped using confronting two-pair primers PCR (PCR-CTPP). The primers were F1: TTT GAT GGA CCT CTG AAT CTC, R1: TTT TCT CAA TGA TAA CAT CCA CT*C*, F2: CTT GAC CAC ATT TTG GGA AC, and R2: TTC CAA TTT CTC TAT ATC CTT GAT GA. An initial 15 minute denaturation at 95° was followed by 40 cycles of 1 min at 64°C, 1 min at 72°C, and 35 s at 95.5°C. The amplified DNA was visualized on a 2% agarose gel with ethidium bromide staining. The amplified DNA was 184 bp for *G *allele, 256 bp for *C *allele, and 397 bp for common band (Figure 1).

#### FAS 1377G>A, FAS 670A>G, FASL 844T>C

**Figure 2:**
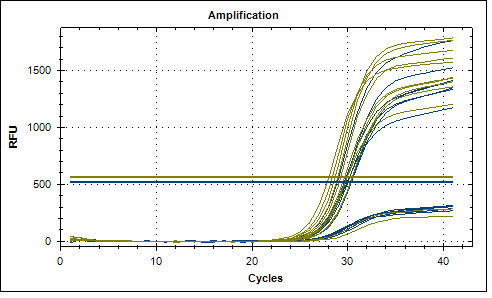
**Gene polymorphisms of *FAS 1377G>A, FAS 670A>G *and *FASL 844T>C *were determined using Taqman genotyping assays with Biorad CFX96™ real-time cycler (Bio-Rad Laboratories Inc, Hercules, USA)**.

SNPs of *FAS 1377G>A *(rs2234767), *FAS 670A>G *(rs1800682) and *FASL 844T>C *(rs763110) were genotyped by using predesigned TaqMan assays with a BioRad CFX96™ real-time cycler, in accordance with the manufacturer's instructions (Bio-Rad Laboratories Inc, Hercules, USA). Thermal cycling conditions for polymerase chain reaction (PCR) were, first, denaturing at 95°C for 10 min, followed by 40 cycles of 95.5°C for 15 s and 60°C for 1 min (Figure 2).

### Statistical analysis

Concordance of genotype frequencies with the Hardy-Weinberg equilibrium was tested by online software http://ihg.gsf.de/cgi-bin/hw/hwa1.pl. Age is shown as means and standard deviations, and was compared using ANOVA and unpaired Student's t-test. Categorical data (e.g. gender, distribution of genotypes or alleles) are presented as frequencies; comparisons were performed using the Chi-square test. Association between HRAG and GC with gene polymorphisms were calculated using unconditional multiple logistic regression analysis and expressed as odds ratios (OR) with 95% confidence intervals (CI). The ORs presented in the study were not adjusted for confounding factors. The relative risks for mutations were studied using recessive and dominant model that led to a comparison between wildtype+heterozygous vs. homozygous and wildtype *vs*. heterozygous+homozygous, respectively. Statistical analysis was done using statistical software SPSS Version 16.0 (SPSS Inc., Chicago, Illinois, USA).

## Results

The characteristics of the study groups are presented in Table 1. German and Baltic study groups did not differ significantly according to the allele distribution (data not shown), therefore the respective groups with HRAG as well as controls were combined and further analyzed together. Patients differed significantly according to age and gender distribution. Male made up for almost 2/3 of patients with GC, while both other groups demonstrated inverse findings. Furthermore, controls were significantly younger (6 or 9 years) than both other groups (Table 1). Notably, serological *H. pylori *status were similar among the three groups, the proportion of *H. pylori*-positive subjects was between 59-62%, and did not differ significantly.

**Table 1:** Characteristics of groups

	Controls (n = 238)	GC (n = 114)	HRAG (n = 222)	ANOVA (Age) Chi-squared test *p *value
Age				
Mean ± SD	56.9 ± 16.1	65.51 ± 13.4	63.4 ± 10.4	< 0.001
Gender				
Male	79 (33.2%)	77 (67.5%)	87 (39.2%)	< 0.001
Female	159 (66.8%)	37 (32.5%)	135 (60.8%)	
*H. pylori*				
Positive	141 (59.2%)	70 (61.4%)	133 (59.9%)	0.928
Negative	97 (40.8%)	44 (38.6%)	89 (40.1%)	

Statistical analysis was performed globally for all three groups.

GC, gastric cancer; HRAG, high risk atrophic gastritis

All individuals were successfully genotyped for *ACE I/D, FAS 1377G>A, FAS 670A>G *and *FASL 844T>C *polymorphisms. Four subjects were not genotyped for *NOD1 796G>A *polymorphism and five subjects for *TLR4 3725G>C *polymorphism. The genotype distributions for all six polymorphisms in control, HRAG and GC groups were similar to those expected for Hardy-Weinberg equilibrium (data not shown). The genotype frequencies of *ACE I/D, NOD1 796G>A, TLR4 3725G>C, FAS 1377G>A, FAS 670A>G *and *FASL 844T>C *among different groups are presented in Table 2. Analysis of genotypes and allele frequencies showed similar distribution between GC, HRAG, and controls. We did not find any significant association between all polymorphisms included in our study with respect to GC and HRAG. We also performed an analysis comparing control group with a risk group (combined GC and HRAG subjects); however it did not reveal significant differences in genotype frequencies (Additional file 1, **Table S1**).

**Table 2:** Association of *ACE, NOD1, TLR4, FAS *and *FASL *gene polymorphisms with gastric cancer and high risk atrophic gastritis

Genotypes	Controls (n = 238)	GC (n = 114)			HRAG (n = 222)		
	
	n (%)	n (%)	OR (95% CI)	*p*	n (%)	OR (95% CI)	*p*
*ACE I/D*							
*I/I*	62 (26.1)	27 (23.7)	0.88 (0.52-1.48)	0.632	62 (27.9)	1.10 (0.73-1.66)	0.650
*I/D*	110 (46.2)	59 (51.8)	1.25 (0.79-1.95)	0.331	108 (48.6)	1.10 (0.76-1.59)	0.602
*D/D*	66 (27.7)	28 (24.6)	0.85 (0.51-1.42)	0.529	52 (23.4)	0.79 (0.52-1.21)	0.290
*Allele I*	234 (49.2)	113 (49.6)	1.02 (0.74-1.39)	0.921	232 (52.3)	1.13 (0.87-1.47)	0.348
*Allele D*	242 (50.8)	115 (50.4)	0.98 (0.72-1.35)	0.921	212 (47.7)	0.88 (0.68-1.15)	0.348
							
*NOD1 796G>A*							
*G/G*	129 (54.4)	61 (54.5)	1.00 (0.64-1.57)	0.995	138 (62.4)	1.39 (0.96-2.02)	0.082
*G/A*	85 (35.9)	40 (35.7)	0.99 (0.62-1.59)	1.000	66 (29.9)	0.76 (0.52-1.13)	0.172
*A/A*	23 (9.7)	11 (9.8)	1.01 (0.48-2.16)	0.973	17 (7.7)	0.78 (0.40-1.49)	0.446
*Allele G*	343 (72.4)	162 (72.3)	0.99 (0.70-1.42)	0.991	342 (77.4)	1.31 (0.97-1.76)	0.081
*Allele A*	131 (27.6)	62 (27.7)	1.00 (0.70-1.73)	0.991	100 (22.6)	0.77 (0.57-1.03)	0.081
							
*TLR4 3725G>C*							
*G/G*	190 (80.5)	90 (79.6)	0.95 (0.54-1.66)	0.849	181 (82.3)	1.12 (0.70-1.80)	0.629
*G/C*	41 (17.4)	21 (18.6)	1.09 (0.61-1.94)	0.782	33 (15.0)	0.84 (0.51-1.38)	0.492
*C/C*	5 (2.1)	2 (1.8)	0.83 (0.16-4.36)	0.832	6 (2.7)	1.29 (0.39-4.31)	0.672
*Allele G*	421 (89.2)	201 (88.9)	0.97 (0.59-1.62)	0.919	395 (89.8)	1.06 (0.69-1.62)	0.776
*Allele C*	51 (10.8)	25 (11.1)	1.03 (0.62-1.71)	0.919	45 (10.2)	0.94 (0.62-1.44)	0.776
							
*FAS 1377G>A*							
*G/G*	197 (82.8)	95 (83.3)	1.04 (0.57-1.88)	0.895	178 (80.2)	0.84 (0.52-1.35)	0.471
*G/A*	40 (16.8)	18 (15.8)	0.92 (0.51-1.70)	0.809	41 (18.5)	1.12 (0.69-1.81)	0.640
*A/A*	1 (0.4)	1 (0.9)	2.09 (0.13-33.8)	0.593	3 (1.4)	3.2 (0.33-31.4)	0.282
*Allele G*	434 (91.2)	208 (91.2)	1.00 (0.57-1.75)	0.981	397 (89.4)	0.82 (0.52-1.27)	0.366
*Allele A*	42 (8.8)	20 (8.8)	0.99 (0.56-1.73)	0.981	47 (10.6)	1.22 (0.79-1.90)	0.366
							
*FAS 670A>G*							
*A/A*	70 (29.4)	31 (27.2)	0.89 (0.54-1.47)	0.666	68 (30.6)	1.06 (0.71-1.57)	0.775
*A/G*	127 (53.4)	62 (54.4)	1.04 (0.66-1.63)	0.856	101 (45.5)	0.72 (0.51-1.05)	0.091
*G/G*	41 (17.2)	21 (18.4)	1.08 (0.60-1.94)	0.783	53 (23.9)	1.50 (0.95-2.39)	0.077
*Allele A*	267 (56.1)	124 (54.4)	0.93 (0.68-1.28)	0.699	237 (53.4)	0.89 (0.69-1.16)	0.408
*Allele G*	209 (43.9)	104 (45.6)	1.07 (0.78-1.47)	0.699	207 (46.6)	1.12 (0.86-1.44)	0.408
							
*FASL 844T>C*							
*T/T*	124 (52.1)	55 (48.2)	0.86 (0.55-1.34)	0.498	108 (48.6)	0.87 (0.60-1.25)	0.459
*T/C*	94 (39.5)	52 (45.6)	1.28 (0.81-2.01)	0.275	91 (41.0)	1.06 (0.73-1.54)	0.743
*C/C*	20 (8.4)	7 (6.1)	0.71 (0.29-1.73)	0.455	23 (10.4)	1.26 (0.67-2.36)	0.471
*Allele T*	342 (71.8)	162 (71.1)	0.96 (0.67-1.36)	0.826	307 (69.1)	0.87 (0.66-1.17)	0.368
*Allele C*	134 (28.2)	66 (28.9)	1.04 (0.73-1.47)	0.826	137 (30.9)	1.14 (0.86-1.51)	0.368

GC, gastric cancer; HRAG, high risk atrophic gastritis, CI, confidence interval; OR, odds ratio The ORs were calculated comparing each genotype *vs*. the other two genotypes, the first line for each gene polymorphism represents the dominant model and third line represents the recessive model.

We observed a tendency for *NOD1 796G/G *genotype for increased risk of atrophic gastritis (62.4% *vs*. 54.5% in controls, *p *= 0.082). Similarly, *NOD1 *allele *G *was more common in patients with HRAG (77.4%) when compared to controls (72.4%), but without significance (*p *= 0.081). *FAS 670G/G *genotype was more frequent in HRAG group when compared to controls, 23.9% and 17.2% respectively, however it failed to reach significance level (*p *= 0.077). The *FAS 670G/A *genotype was less frequent in HRAG (45.5%) when compared to controls (53.4%), but the difference was not statistically significant (*p *= 0.091).

We also analyzed the distribution of genotypes with respect to different histological GC types (Additional file 2, **Table S2**). Histological type of GC was intestinal, diffuse and mixed type in 47, 47 and 20 patients, respectively. In order to compare intestinal and diffuse type GC, mixed tumor types were excluded from subanalysis. *ACE D/D *polymorphism was found to be less prevalent in diffuse type GC, when compared to controls (27.7% *vs*. 12.8%, respectively; *OR*-0.38, 95% *CI *0.15-0.94, *p *= 0.030). There were no differences between different histological subtypes of GC with respect to the other gene polymorphisms. We also analyzed the seropositivity status of *H. pylori *with respect to *TLR4 3725G>C *and *NOD1 796G>A *polymorphisms, but no significant differences in genotype distribution were observed (Table 3).

**Table 3:** Distribution of *NOD1 *and *TLR4 *gene polymorphisms in *Helicobacter pylori *positive and negative subjects

Genotypes	*Hp *negative	*Hp *positive		
	**n (%)**	**n (%)**	**OR (95% CI)**	***p***

*NOD1 796G>A*				
*G/G*	133 (57.8)	195 (57.4)	0.98 (0.69-1.37)	0.910
*G/A*	75 (32.6)	116 (34.1)	1.07 (0.75-1.52)	0.708
*A/A*	22 (9.60)	29 (8.50)	0.88 (0.49-1.57)	0.670
*Allele G*	341 (74.1)	506 (74.4)	1.01 (0.77-1.33)	0.915
*Allele A*	119 (25.9)	174 (25.6)	0.98 (0.75-1.29)	0.915
				
*TLR4 3725G>C*				
*G/G*	182 (79.1)	279 (82.3)	1.22 (0.80-1.87)	0.343
*G/C*	44 (19.1)	51 (15.0)	0.74 (0.48-1.16)	0.199
*C/C*	4 (1.8)	9 (2.7)	1.54 (0.46-5.06)	0.549
*Allele G*	408 (88.7)	609 (89.8)	1.12 (0.76-1.64)	0.545
*Allele C*	52 (11.3)	69 (10.2)	0.88 (0.60-1.30)	0.545

*Hp*, *Helicobacter pylori; *OR, odds ratio

The ORs were calculated comparing each genotype *vs*. the other two genotypes, the first line for each gene polymorphism represents the dominant model and third line represents the recessive model.

## Discussion

Overall, in our study *ACE I/D, NOD1 796G>A, TLR4 3725G>C, FAS 1377G>A, FAS 670A>G *and *FASL 844T>C *gene polymorphisms were not associated with the presence of GC or HRAG. Here, we evaluated six genetic polymorphisms related to the immune-recognition of *H. pylori*, proliferation and apoptosis that were previously described to be associated with increased risk of GC or premalignant gastric lesions in different case-control studies; however reported data are partially conflicting or just based on one study [11-14,20,21,26,33-35]. Since *ACE, NOD1, TLR4, FAS*, and *FASL *have been shown to be involved in gastric carcinogenesis pathways, we expected that the polymorphisms of genes encoding these proteins could be related to GC.

When analyzing *ACE I/D *polymorphism we hypothesized that carriers of *D *allele, which was associated with higher *ACE *activity [10], could have an increased risk of GC. Ebert *et al*. [11] identified an association of *ACE D/D *genotype with the development of early GC, and the same group of researchers showed that this genotype is related to the number of metastatic lymph nodes in GC, but not to the overall risk for GC in German subjects [12]. A Japanese study did not find an association between *ACE I/D *polymorphism and suseptibility to GC [13]. Another Asian study [14] reported that *ACE I/D *polymorphism carried a higher risk for GC with odds ratio of 1.59, however the same study did not find significant association of *ACE *polymorhisms with gastric atrophy. *ACE I/D *genotype frequencies in our study are in line with previous reports [12]. We did not observe significant link between *ACE I/D *polymorphisms and the risk for GC or HRAG.

Considering the relationship between *H. pylori*-induced chronic inflammation and carcinogenesis in the stomach it was tempting to speculate that genetic variation in *NOD1 *receptor, which is involved in bacterial recognition, could be associated with *H. pylori*-induced diseases. *NOD1 796A/A *homozygous mutants were linked with increased risk for peptic ulcer disease in a Hungarian study [20]; however they did not find a significant association with atrophic gastritis. A recent study from Turkey [21] found that subjects with *NOD1 796A/A *genotype had a significantly increased risk for gastric atrophy and antral intestinal metaplasia with very high odds ratios, 34.2 and 39.7 respectively. Such a strong association reported by Kara *et al*. [21] urged us to evaluate the possible association of this polymorphism in our subjects with GC and HRAG. The genotype frequencies of *NOD1 796G>A *in this study correspond to previous reports [20,21]. In our study we observed a tendency for *NOD1 796G/G *genotype and allele *G *for increased risk for atrophic gastritis; however the difference did not reach statistical significance. We did not observe an association between *NOD1 796G>A *gene polymorphism and HRAG or GC. *H. pylori *seropositivity status was also not linked with *NOD1 796G>A *genotypes.

Polymorphisms of *TLR4 *gene are hypothesized to decrease responsiveness to gram-negative bacteria lipopolysaccharide through alterations in binding. In a recent study *TLR4 3725G>C *polymorphism was indentified as a risk factor for severe gastric atrophy in *H. pylori *sero-positive Japanese subjects with OR of 1.43 and 1.47 for *G/C *and *C/C *genotypes, respectively [26], however in the same study this polymorphism was not associated with the risk for GC. Another study showed a combined effect of *TLR4 3725G>C *and *miR-146a G>C *gene polymorphisms for risk of gastric atrophy, but not for GC [43]. Our data on *TLR4 3725G>C *genotype frequencies are comparable to the reported frequencies in leukemia study on Caucasian population [44]. We did not find significant association between *TLR4 3725G>C *genotypes and HRAG or GC. There was also no association between *H. pylori *seropositivity and *TLR4 3725G>C *genotypes.

There are only few studies that have evaluated the risk for GC in relation to *FAS *and *FASL *gene polymorphisms. Reported results are conflicting and cover Asian subjects only. In two Chinese case-control studies *FAS *and *FASL *genotypes had no significant associations with risk of GC [33,34]. Another study from China suggested that *FASL 844T/T *or *T/C *and *FAS 1377A/A *genotypes could be a risk factor for GC in combination with other gene polymorphisms [32]. Hsu *et al*. [35] have reported that *FAS 1377 *allele *A *was a protective factor for developing intestinal metaplasia in the antrum with odds ratio 0.3, while carrying the *FASL 844 *allele *C *was a risk factor for developing gastric atrophy in the corpus with OR of 9.4. The distribution of FAS and FASL genotypes in our cohorts corresponds to the frequencies reported on Caucasian subjects in a lung cancer study [45], but we could not provide any evidence that *FAS *and *FASL *gene polymorphism are linked with risk for GC or HRAG in Caucasians.

In the present study we evaluated potential links between the risk for GC and several carcinogenesis-related gene polymorphisms that have been rarely or not described, especially in Caucasian population. We did not find significant associations between the presence of GC or HRAG and *ACE I/D, NOD1 796G>A, TLR4 3725G>C, FAS 1377G>A, 670A>G *and *FASL 844T>C *gene polymorphisms. We also analyzed the genotype frequencies with respect to different histological GC types. *ACE D/D *genotype was less prevalent in diffuse-type GC than in controls, however the corresponding subgroups of intestinal and diffuse-type GC are rather small are therefore firm conclusions can not be drawn. Because the studies on *ACE I/D, NOD1 796G>A, TLR4 3725G>C, FAS 1377G>A, 670A>G *and *FASL 844T>C *polymorphisms remain limited, evaluation of the association with GC and premalignant gastric lesions requires additional research. Since the strength of the association may depend on the studied population, larger studies of different ethnic groups with different genetic profiles are required. The differences in current data on these polymorphisms may result from study design, *H. pylori *prevalence, and different histological subtypes of GC. In this study there was no difference in *H. pylori *positivity among GC, HRAG and control groups. The possible explanation for these findings could be higher prevalence of *H. pylori *in Baltic countries, when compared to Germany. HRAG and control groups were selected both in Germany and Baltic countries, while GC subjects were recruited only in the German centre, thus possibly affected the *H. pylori *status within the groups. Alterations in various genes, including oncogenes, tumor-suppressor genes, proinflammatory genes, bacterial recognition and cell-adhesion-related genes have been studied in gastric carcinogenesis [5,24,46]. In previous studies we also evaluated the role of *IL-1B, IL-1RN *and *NOD2 *gene polymorphisms with respect to risks for GC; however no significant associations were identified [36-38]. Some reports suggested that host genetic factors determine the severity of gastric damage and the eventual clinical outcome of *H. pylori *infection [47,48]. These findings however have not been transferred to daily clinical practice, and therefore applicable predisposing genetic factors remain still to be determined.

## Conclusions

The study shows that the polymorphisms of *ACE, NOD1, TLR4, FAS *and *FASL *genes are not associated with *H. pylori*-induced premalignant gastric conditions and GC in subjects of Caucasian ethnicity. Based on the data available now, the investigated polymorphisms are not applicable for identifying individuals with higher risk for developing GC.

## Abbreviations

GC: gastric cancer; HRAG: high risk atrophic gastritis; OR: odds ratio; CI: confidence interval; ACE: Angiotensin converting enzyme; NOD1: Nod-like receptor 1; TLR4: Toll-like receptor 4; *H. pylori*: *Helicobacter pylori*; AG: atrophic gastritis; IM: intestinal metaplasia; RFLP: restriction fragment length polymorphism analysis; SNPs: single nucleotide polymorphisms.

## Competing interests

The authors declare that they have no competing interests.

## Authors' contributions

JK performed acquisition of data, SNP genotyping, statistical analysis, drafted the manuscript; TW was involved in study design, coordination, drafting and final revision of the manuscript; JB, MS, EJ, GK, LJ performed acquisition of data; ML was involved in study design and data collection; PM was involved in study design, and final revision of the manuscript. All authors read and approved the final manuscript.

## Pre-publication history

The pre-publication history for this paper can be accessed here:

http://www.biomedcentral.com/1471-2350/12/112/prepub

## Supplementary Material

Additional file 1 **Table S1**. Distribution of *ACE, NOD1, TLR4, FAS *and *FASL *gene polymorphisms in control and risk group (gastric cancer and high risk atrophic gastritis patients). Click here for file

Additional file 2 **Table S2**. Distribution of *ACE, NOD1, TLR4, FAS *and *FASL *gene polymorphisms in controls, intestinal and diffuse type gastric cancer groups. Click here for file
