# Supplementary material for: Lack of association between gene polymorphisms of Angiotensin converting enzyme, Nod-like receptor 1, Toll-like receptor 4, FAS/FASL and the presence of Helicobacter pylori-induced premalignant gastric lesions and gastric cancer in Caucasians
Source: BMC Med Genet. 2011 Aug 24;12:112. doi: 10.1186/1471-2350-12-112 (PMC3166912; doi:10.1186/1471-2350-12-112)
Supplement: Additional file 1 — Table S1. Distribution of ACE, NOD1, TLR4, FAS and FASL gene polymorphisms in control and risk group (gastric cancer and high risk atrophic gastritis patients). [file 1471-2350-12-112-S1.DOC]

**Additional file 1, Table S1.** Distribution of *ACE, NOD1, TLR4, FAS* and *FASL* gene polymorphisms in control and risk group (gastric cancer and high risk atrophic gastritis patients).

| Genotypes | Controls | GC + HRAG |  |  |
| --- | --- | --- | --- | --- |
|  | n (%) | n (%) | OR (95% CI) | *p* |
| *ACE I/D* |  |  |  |  |
| *I/I* | 62 (26.1) | 89 (26.5) | 1.02 (0.70-1.49) | 0.907 |
| *I/D* | 110 (46.2) | 167 (49.7) | 1.14 (0.82-1.60) | 0.410 |
| *D/D* | 66 (27.7) | 80 (23.8) | 0.81 (0.56-1.19) | 0.288 |
| *Allele I* | 234 (49.2) | 345 (51.3) | 1.09 (0.86-1.38) | 0.466 |
| *Allele D* | 242 (50.8) | 327 (48.7) | 0.91 (0.72-1.16) | 0.466 |
|  |  |  |  |  |
| *NOD1 796G>A* |  |  |  |  |
| *G/G* | 129 (54.4) | 199 (59.8) | 1.24 (0.88-1.74) | 0.204 |
| *G/A* | 85 (35.9) | 106 (31.8) | 0.83 (0.58-1.18) | 0.314 |
| *A/A* | 23 (9.70) | 28 (8.40) | 0.85 (0.48-1.52) | 0.593 |
| *Allele G* | 343 (72.4) | 504 (75.6) | 1.18 (0.90-1.55) | 0.207 |
| *Allele A* | 131 (27.6) | 162 (24.4) | 0.84 (0.64-1.10) | 0.207 |
|  |  |  |  |  |
| *TLR4 3725G>C* |  |  |  |  |
| *G/G* | 190 (80.5) | 271 (81.4) | 1.05 (0.69-2.76) | 0.793 |
| *G/C* | 41 (17.4) | 54 (16.2) | 0.92 (0.59-1.43) | 0.715 |
| *C/C* | 5 (2.1) | 8 (2.4) | 1.13 (0.36-3.50) | 0.823 |
| *Allele G* | 421 (89.2) | 596 (89.5) | 1.03 (0.70-1.51) | 0.873 |
| *Allele C* | 51 (10.8) | 70 (10.5) | 0.97 (0.66-1.42) | 0.873 |
|  |  |  |  |  |
| *FAS 1377G>A* |  |  |  |  |
| *G/G* | 197 (82.8) | 273 (81.2) | 0.90 (0.58-1.39) | 0.640 |
| *G/A* | 40 (16.8) | 59 (17.6) | 1.05 (0.67-1.63) | 0.814 |
| *A/A* | 1 (0.4) | 4 (1.2) | 2.85 (0.32-25.7) | 0.327 |
| *Allele G* | 434 (91.2) | 605 (90.0) | 0.87 (0.58-1.31) | 0.513 |
| *Allele A* | 42 (8.8) | 67 (10.0) | 1.14 (0.76-1.71) | 0.513 |
|  |  |  |  |  |
| *FAS 670A>G* |  |  |  |  |
| *A/A* | 70 (29.4) | 99 (29.5) | 1.00 (0.69-1.44) | 0.989 |
| *A/G* | 127 (53.4) | 163 (48.5) | 0.82 (0.59-1.14) | 0.252 |
| *G/G* | 41 (17.2) | 74 (22.0) | 1.35 (0.88-2.07) | 0.157 |
| *Allele A* | 267 (56.1) | 361 (53.7) | 0.90 (0.72-1.15) | 0.426 |
| *Allele G* | 209 (43.9) | 311 (46.3) | 1.10 (0.86-1.39) | 0.426 |
|  |  |  |  |  |
| *FASL 844T>C* |  |  |  |  |
| *T/T* | 124 (52.1) | 163 (48.5) | 0.86 (0.62-1.21) | 0.396 |
| *T/C* | 94 (39.5) | 143 (42.6) | 1.13 (0.80-1.59) | 0.462 |
| *C/C* | 20 (8.4) | 30 (8.9) | 1.07 (0.59-1.93) | 0.826 |
| *Allele T* | 342 (71.8) | 469 (69.8) | 0.91 (0.69-1.17) | 0.450 |
| *Allele C* | 134 (28.2) | 203 (30.2) | 1.10 (0.85-1.43) | 0.450 |

GC, gastric cancer; HRAG, high risk atrophic gastritis; OD, odds ratio

The ORs were calculated comparing each genotype *vs.* the other two genotypes, the first line for each gene polymorphism represents the dominant model and third line represents the recessive model.
