# Supplementary material for: Lack of association between gene polymorphisms of Angiotensin converting enzyme, Nod-like receptor 1, Toll-like receptor 4, FAS/FASL and the presence of Helicobacter pylori-induced premalignant gastric lesions and gastric cancer in Caucasians
Source: BMC Med Genet. 2011 Aug 24;12:112. doi: 10.1186/1471-2350-12-112 (PMC3166912; doi:10.1186/1471-2350-12-112)
Supplement: Additional file 2 — Table S2. Distribution of ACE, NOD1, TLR4, FAS and FASL gene polymorphisms in controls, intestinal and diffuse type gastric cancer groups. [file 1471-2350-12-112-S2.DOC]

**Additional file 2, Table S2.** Distribution of *ACE, NOD1, TLR4, FAS* and *FASL* gene polymorphisms in controls, intestinal and diffuse type gastric cancer groups.

| Genotypes | Controls (n=238) | Intestinal type GC  (n=47) | | | Diffuse type GC  (n=47) | | |
| --- | --- | --- | --- | --- | --- | --- | --- |
|  | n (%) | n (%) | OR (95% CI) | *p* | n (%) | OR (95% CI) | *p* |
| *ACE I/D* |  |  |  |  |  |  |  |
| *I/I* | 62 (26.1) | 8 (17.0) | 0.58 (0.25-1.31) | 0.188 | 14 (29.8) | 1.20 (0.60-2.39) | 0.596 |
| *I/D* | 110 (46.2) | 21 (44.7) | 0.93 (0.50-1.76) | 0.846 | 27 (57.4) | 1.57 (0.83-2.95) | 0.159 |
| *D/D* | 66 (27.7) | 18 (38.3) | 1.61 (0.84-3.10) | 0.146 | 6 (12.8) | 0.38 (0.15-0.94) | 0.030 |
| *Allele I* | 234 (49.2) | 37 (39.4) | 0.67 (0.42-1.05) | 0.082 | 55 (58.5) | 1.45 (0.93-2.28) | 0.097 |
| *Allele D* | 242 (50.8) | 57 (60.6) | 1.49 (0.94-2.33) | 0.082 | 39 (41.5) | 0.68 (0.43-1.07) | 0.097 |
|  |  |  |  |  |  |  |  |
| *NOD1 796G>A* |  |  |  |  |  |  |  |
| *G/G* | 129 (54.4) | 26 (55.4) | 1.03 (0.55-1.94) | 0.910 | 31 (66.0) | 1.62 (0.84-3.12) | 0.145 |
| *G/A* | 85 (35.9) | 16 (34.0) | 0.92 (0.47-1.78) | 0.811 | 12 (25.5) | 0.61 (0.30-1.24) | 0.172 |
| *A/A* | 23 (9.7) | 5 (10.6) | 1.10 (0.39-3.07) | 0.844 | 4 (8.5) | 0.86 (0.28-2.62) | 0.798 |
| *Allele G* | 343 (72.4) | 68 (72.3) | 0.99 (0.60-1.63) | 0.996 | 74 (78.7) | 1.41 (0.82-2.40) | 0.202 |
| *Allele A* | 131 (27.6) | 26 (27.7) | 1.00 (0.61-1.64) | 0.996 | 20 (21.3) | 0.70 (0.41-1.20) | 0.202 |
|  |  |  |  |  |  |  |  |
| *TLR4 3725G>C* |  |  |  |  |  |  |  |
| *G/G* | 190 (80.5) | 38 (80.9) | 1.02 (0.46-2.26) | 0.956 | 36 (76.6) | 0.79 (0.37-1.67) | 0.541 |
| *G/C* | 41 (17.4) | 8 (17.0) | 0.97 (0.42-2.24) | 1.000 | 10 (21.3) | 1.28 (0.59-2.79) | 0.524 |
| *C/C* | 5 (2.1) | 1 (2.1) | 1.00 (0.11-8.79) | 0.996 | 1 (2.1) | 1.00 (0.11-8.79) | 0.996 |
| *Allele G* | 421 (89.2) | 84 (89.4) | 1.01 (0.49-2.08) | 0.962 | 82 (87.2) | 0.82 (0.42-1.62) | 0.580 |
| *Allele C* | 51 (10.8) | 10 (10.6) | 0.98 (0.48-2.01) | 0.962 | 12 (12.8) | 1.20 (0.61-2.36) | 0.580 |
|  |  |  |  |  |  |  |  |
| *FASL 1377G>A* |  |  |  |  |  |  |  |
| *G/G* | 197 (82.8) | 37 (78.7) | 0.77 (0.35-1.67) | 0.508 | 43 (91.5) | 2.23 (0.76-6.57) | 0.134 |
| *G/A* | 40 (16.8) | 9 (19.1) | 1.17 (0.52-2.61) | 0.697 | 4 (8.5) | 0.46 (0.15-1.35) | 0.150 |
| *A/A* | 1 (0.4) | 1 (2.1) | 5.15 (0.31-83.81) | 0.200 | 0 (0.0) | 1.66 (0.06-41.5) | 0.656 |
| *Allele G* | 434 (91.2) | 83 (88.3) | 0.73 (0.36-1.47) | 0.379 | 90 (95.7) | 2.17 (0.76-6.22) | 0.137 |
| *Allele A* | 42 (8.8) | 11 (11.7) | 1.36 (0.67-2.76) | 0.379 | 4 (4.3) | 0.45 (0.16-1.31) | 0.137 |
|  |  |  |  |  |  |  |  |
| *FAS 670A>G* |  |  |  |  |  |  |  |
| *A/A* | 70 (29.4) | 9 (19.1) | 0.56 (0.26-1.23) | 0.150 | 13 (27.7) | 0.91 (0.45-1.84) | 0.809 |
| *A/G* | 127 (53.4) | 28 (59.6) | 1.28 (0.68-2.43) | 0.434 | 27 (57.4) | 1.17 (0.62-2.21) | 0.607) |
| *G/G* | 41 (17.2) | 10 (21.3) | 1.29 (0.59-2.82) | 0.508 | 7 (14.9) | 0.84 (0.35-2.00) | 0.696 |
| *Allele A* | 267 (56.1) | 46 (48.9) | 0.75 (0.48-1.16) | 0.202 | 53 (56.4) | 1.01 (0.64-1.58) | 0.958 |
| *Allele G* | 209 (43.9) | 48 (51.1) | 1.33 (0.85-2.07) | 0.202 | 41 (43.6) | 0.98 (0.63-1.54) | 0.958 |
|  |  |  |  |  |  |  |  |
| *FASL 844T>C* |  |  |  |  |  |  |  |
| *T/T* | 124 (52.1) | 22 (46.8) | 0.80 (0.43-1.51) | 0.507 | 23 (48.9) | 0.88 (0.47-1.64) | 0.691 |
| *T/C* | 94 (39.5) | 24 (51.1) | 1.59 (0.85-2.99) | 0.141 | 21 (44.7) | 1.23 (0.65-2.32) | 0.507 |
| *C/C* | 20 (8.4) | 1 (2.1) | 0.23 (0.03-1.81) | 0.132 | 3 (6.4) | 0.74 (0.21-2.61) | 0.642 |
| *Allele T* | 342 (71.8) | 68 (72.3) | 1.02 (0.62-1.68) | 0.922 | 67 (71.3) | 0.97 (0.59-1.58) | 0.910 |
| *Allele C* | 134 (28.2) | 26 (27.7) | 0.97 (0.59-1.59) | 0.922 | 27 (28.7) | 1.02 (0.63-1.67) | 0.910 |

GC, gastric cancer; OD, odds ratio

The ORs were calculated comparing each genotype *vs.* the other two genotypes, the first line for each gene polymorphism represents the dominant model and third line represents the recessive model.
